# Supplementary material for: The Construction and Exploration of a Comprehensive MicroRNA Centered Regulatory Network in Foxtail Millet (Setaria italica L.)
Source: Front Plant Sci. 2022 May 6;13:848474. doi: 10.3389/fpls.2022.848474 (PMC9121102; doi:10.3389/fpls.2022.848474)

T=Seita.2G192700.1_Q=Sit−miRN2548_S=2400

category=2_p=0.999999999999986

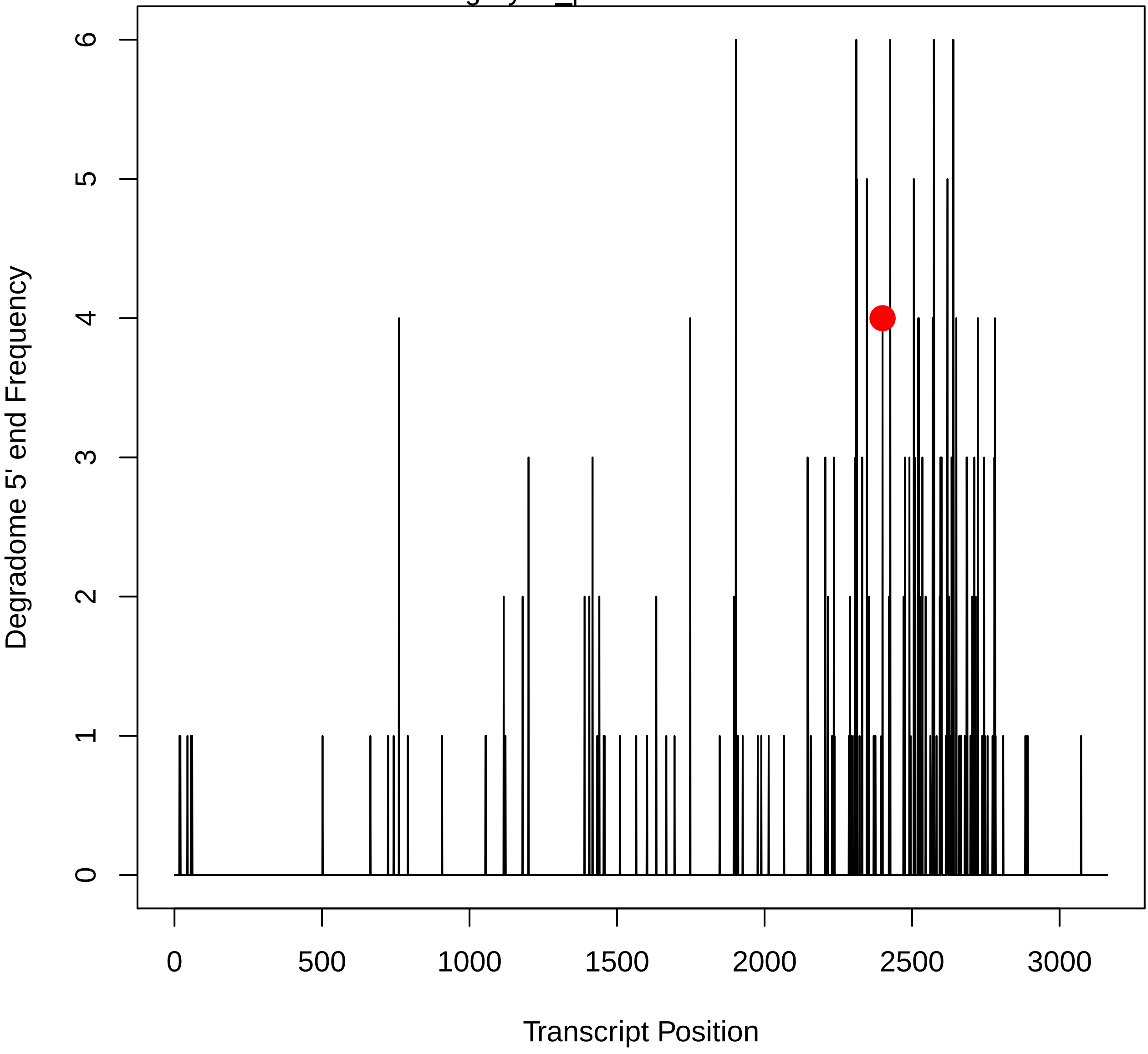

Supplement: Supplementary file 3 [file Data_Sheet_3.zip › Sit-miRN2548_Seita.2G192700.1_2400_TPlot.pdf]
